# Acoustic-based detection of debonding in thin-layer semi-rigid base asphalt pavements using a-weighted sound pressure levels

**DOI:** 10.1371/journal.pone.0334910

**Published:** 2025-10-30

**Authors:** Changfeng Hao, Min Ye, Qing Zhang, Zhongyu Li, Xianbao Zuo, Zhen Sun

**Affiliations:** 1 National Engineering Research Center of Highway Maintenance Equipment, Chang’an University, Xi’an, Shaanxi, China; 2 Henan Gaoyuan Highway Maintenance Technology Co., LTD, Xinxiang, Henan, China; Shandong University of Technology, CHINA

## Abstract

Thin-layer interlayer debonding in semi-rigid base asphalt pavements is difficult to detect with conventional techniques due to their limited sensitivity. This study proposes a novel acoustic-based detection approach that utilizes a distributed elastic variable damping (DEVD) Maxwell model and introduces an A-weighted sound pressure level (SPL) index to enhance detection of small-scale debonding. Comprehensive numerical simulations were integrated with full-scale experiments to analyze acoustic signals under different excitation modes and debonding scenarios. The results show a strong correlation between the A-weighted acoustic metrics and debonding area, and the proposed method reliably distinguishes thin debonding layers that ground-penetrating radar (GPR) could not detect. Our acoustic method demonstrated superior sensitivity to thin-layer debonding, highlighting its potential as a non-destructive tool for early pavement damage detection and improved road maintenance.

## Introduction

Semi-rigid base asphalt pavement is a commonly adopted structural form in highway construction throughout China, designed to bear substantial traffic loads [1,2]. Over time, factors such as uneven subgrade settlement, water infiltration, and temperature-induced expansion and contraction cause stress redistribution within the asphalt pavement layers. These processes often result in structural discontinuities, manifesting as debonding between layers [3,4]. While these debonding defects are frequently concealed, they can lead to localized excessive bearing pressures, ultimately causing pavement bending, subsidence, reduced structural stability, diminished performance, and a shortened service life [5,6]. Therefore, accurately evaluating the debonding behavior of semi-rigid base asphalt pavement is critical for addressing these issues and ensuring reliable assessments.

Prior studies have advanced debonding detection in Semi-rigid base asphalt pavements and optimized multiple techniques [7,8–10]. Mainstream approaches include ground-penetrating radar (GPR), deflection-based methods (e.g., Traffic Speed Deflectometer, TSD), and acoustic/vibration analysis.

GPR and acoustic/vibration sensing are complementary for interlayer assessment. GPR sensitivity is fundamentally constrained by wavelength and dielectric contrast—very thin or weakly bonded interfaces may be indistinguishable from noise or neighboring reflections even with advanced processing [11–13]. TSD provide basin-shape/deflection information but show limited sensitivity to localized, small-scale debonding under realistic noise and resolution bounds [14–18].The acoustic vibration method detects pavement debonding by analyzing acoustic signals from surface impacts, identifying changes in fatigue, cracks, and debonding [19–21]. It is a non-destructive technique sensitive to early pavement distress [20,22]. However, applying this method to semi-rigid base asphalt pavements is challenging due to structural variations, energy differences in impacts, and complex time-frequency characteristics, compounded by the heterogeneity of asphalt mixtures [23,24]. Studies show that material fatigue alters acoustic properties, with stiffness degradation linked to time-frequency signal changes, while micro-cracks and debonding scatter and attenuate sound waves, enabling early damage detection [25,26].

Researchers have developed various simulation models to analyze asphalt pavement behavior, particularly for debonding detection. Variation in sound frequency and amplitude have been identified as key indicators, leading to the development of analytical models such as axisymmetric models [27], 3D models [28], and generalized Maxwell models [29]. These models simulate viscoelastic pavement behavior under stress, with Kelvin-Voigt and Maxwell models widely used to describe dynamic loading responses. Recent progress has refined these models. Serra-Aguila [30] applied Prony series to enhance viscoelastic property analysis, while Pinto [31] introduced field-corrected and source-corrected approaches for long-term simulations. Ding [32] utilized a fractional Maxwell model to simulate creep behavior in hydraulic fracturing, highlighting the importance of viscoelasticity in material performance. Liang [33] further optimized the Maxwell model to describe ultra-low creep behavior. Nevertheless, these advancements, existing models face limitations, including high computational demands, extensive storage requirements, and difficulties in handling nonlinear materials, restricting their practical application in large-scale pavement simulations.

Accurate identification of thin-layer interlayer debonding is pivotal because it governs assessments of stiffness degradation, fatigue progression, and crack initiation; misclassification drives either unnecessary interventions or missed early damage, inflating life-cycle costs, and shortening service life. In practice, however, small, or weakly bonded interfaces are hard to resolve: radar resolution is bounded by wavelength and dielectric contrast; deflection-based sensing shows limited sensitivity to localized defects under realistic noise and spatial resolution; and prior acoustic studies often stop at qualitative signatures without a simple, field-ready index tied to full-scale evidence.

These constraints motivate a high-sensitivity, field-oriented acoustic approach that (i) preserves modal fidelity while remaining computationally tractable, (ii) yields a robust, interpretable acoustic index for thin-layer debonding, and (iii) is validated at full scale with a head-to-head GPR comparison. We address this gap by integrating a distributed elastic variable damping (DEVD) Maxwell foundation with acoustic–solid coupling and A-weighted indices in an application-oriented and computationally tractable manner, validated on a full-scale specimen. The novelty lies in this efficient model index pipeline and its demonstrated sensitivity to thin-layer debonding that the GPR benchmark failed to reveal.

The remainder of this study is organized as follows: Section 2 presents the simulation model, experimental setup, excitation scheme, and evaluation indices; Section 3 reports numerical and experimental results and the acoustic–GPR comparison; Section 4 discusses scalability and limitations; Section 5 concludes with future work.

## Methodology

### Overall structure

**Fig 1:**
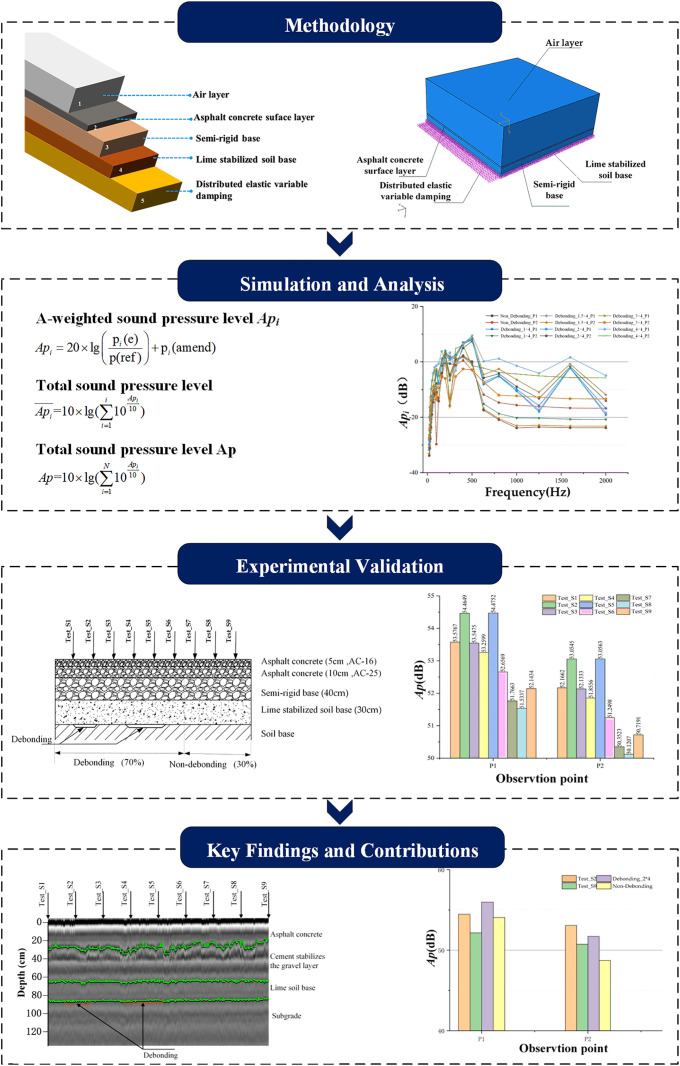
Flow chart of this study.

This study proposes an acoustic-based methodology for detecting interlayer debonding in semi-rigid base asphalt pavements, utilizing the DEVD-Maxwell model to analyze vibration and acoustic signal characteristics. The research framework integrates numerical simulations and experimental validation to establish a correlation between debonding size and acoustic response, providing a novel approach for non-destructive pavement assessment, the flow chart of this study is shown in Fig 1.

### Simulation model

#### Vibration principle.

Given that semi-rigid base asphalt pavement typically has a width of at least 9 m and a thickness of approximately 0.5 m, its thickness is relatively small compared to its surface dimensions—specifically, about 1/18th of the slab width. Moreover, the thickness is negligible relative to the wavelength of the material. As a result, the stress along the thickness direction can be assumed constant, implying that the internal stress depends only on the planar coordinates. However, it can be approximated that all points on the central plane undergo vertical vibrations. Thus, the displacement of the road can be effectively represented by the displacement of the central plane, where η is a function of the planar coordinates and time. Consequently, the vibration of the semi-rigid base asphalt pavement is simplified to a planar plate problem [34,35].

**Fig 2:**
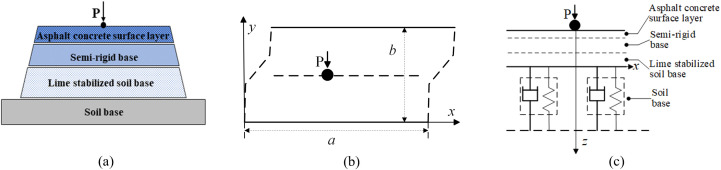
A rectangular plate simply supported on all four edges is employed to represent the semi-rigid base asphalt pavement, which rests on a viscoelastic foundation, (a) pavement structure diagram, (b) location of the excitation point in the x-y coordinate system, (c) excitation transfer model in the x-z coordinate system.

According to the studies by Pérez-Acebo [36] and Zu [37], a four-sided cylinder-supported plate structure is used in this thesis, consisting of the “Asphalt Concrete Surface Layer,” “Semi-Rigid Base,” “Lime Stabilized Soil Base,” and “Soil Base.” Given the similar modulus, Poisson’s ratio, and other parameters of the first three layers, the model is simplified by assigning the same parameters to these layers. The “Soil Base” is adopted as a simplified model for the elastic and damping elements. A rectangular plate simply supported on all four edges is employed to represent the semi-rigid base asphalt pavement, which rests on a viscoelastic foundation [38,39], as shown in Fig 2.

As shown in Fig 2, the governing equation for a rectangular plate simply supported on all four edges (representing the semi-rigid base asphalt pavement) can be written as:


$$D\nabla^{4}w(x,y,t)+\rho h\frac{\partial^{2}w(x,y,t)}{\partial t^{2}}+c\frac{\partial w(x,y,t)}{\partial t}+kw(x,y,t)=P(x,y,t)$$ (1)

where *w*(*x*, *y*, *t*) is transverse displacement of the plate at coordinates (*x*, *y*) and time *t*; $\nabla^{4}=\nabla^{2}(\nabla^{2})$; $\nabla^{2}=\frac{\partial^{2}}{\partial x^{2}}+\frac{\partial^{2}}{\partial y^{2}}$ is the Laplace operator; D=Eh3/Eh312(1−μ2)\nulldelimiterspace12(1−μ2) is the plate columnar bending stiffness; *ρ, h*, *E* and *μ* are the material density, plate thickness, modulus of elasticity and Poisson’s ratio of the plate; *k*, *c* denote the elasticity coefficient and coefficient of viscosity of the foundation, respectively; P(*x*, *y*, *t*) is the vibration load, and a series of sums of the simple harmonic vibration loads can be obtained through the Fourier transform, we assuming a single frequency Ω and the loaded point moves in the direc*t*ion of y = *d* along a straight line at *v*, the load $P(x,y,t)=Pe^{j\Omega t}\delta (x-vtdelta(y-d)$ [40].

For the model shown in Fig 2, the following boundary conditions and initial conditions are assumed:


wx(x,y,t)=∂2w(x,y,t)/∂2w(x,y,t)∂x2\nulldelimiterspace∂x2=0 x∈(0,a)wy(x,y,t)=∂2w(x,y,t)/∂2w(x,y,t)∂y2\nulldelimiterspace∂y2=0 y∈(0,b)wt(x,y,t)=∂2w(x,y,t)/∂2w(x,y,t)∂t\nulldelimiterspace∂t=0 (2)

According to the boundary conditions of Equation (2), the vibration mode function of the dynamic deflection of the model is selected as:


$$w(x,y,t)=\sum_{m=1}^{M}\sum_{n=1}^{N}\Psi_{mn}(t)\sin(A_{m}x)\sin(B_{n}y)$$ (3)

where Am=mπ/mπa\nulldelimiterspacea, Bn=nπ/nπb\nulldelimiterspaceb, *a* is the length of the model and *b* is the width of the model.

Similarly, the load P (x, y, t) can be expressed as:


$$P(x,y,t)=\sum_{m=1}^{M}\sum_{n=1}^{N}P_{mn}(t)\sin\left(A_{m}x\right)\sin\left(B_{n}y\right)$$ (4)

where $\begin{array}{c} P_{mn}(t)=\frac{4Pe^{j\Omega t}}{ab}\sin\left(A_{m}vt\right)\sin\left(B_{n}d\right) \end{array}$.

Transfer both Equation (3) and Equation (4) into Equation (1), the equation in the dynamic deflection function can be obtained:


$$\Psi_{mn}^{\prime \prime }(t)+2H\Psi_{mn}^{\prime }(t)+J^{2}\Psi_{mn}(t)=\frac{P_{mn}(t)}{\rho h}$$ (5)

where H=c/c2ρh\nulldelimiterspace2ρh, J2=Dρh(Am4+2Am2Bn2+Bn4+k/kD\nulldelimiterspaceD).

The solution to Equation (5) can be expressed as the sum of a general solution and a specific solution. The general solution is expressed as follows:


$$\Psi_{mn}(t)=C_{1mn}e^{r_{1}t}+C_{2mn}e^{r_{2}t}$$ (6)

where $r_{1,2}=-H\pm \sqrt{H^{2}-J^{2}}$, C_1*mn*_ and C_2*mn*_ are the generalization coefficients, obtained from the initial conditions of the model.

This leads to the special solution of Equation (5) as:


$${\Psi_{mn}}^{*}(t)=D_{1mn}\cos\left(A_{m}vt\right)+D_{2mn}\sin\left(A_{m}vt\right)$$ (7)

D_1*mn*_ is the coefficient of the cosine component of the m n vibration mode and D_2*mn*_ is the coefficient of the sine component of the m n vibration mode, and these two parameters can be found by bringing Equation (7) into Equation (5), and this parameter is as follows:


$$D_{1mn}=\frac{-8PA_{m}ve^{j\Omega t}(H+j\Omega )\sin\left(B_{n}d\right)}{ab\rho h\left(J^{2}A_{m}{A_{m}}^{*}\right)}$$ (8)

$$D_{2mn}=\frac{4Pe^{j\Omega t}\sin\left(B_{n}d\right)}{ab\rho h\left(JA_{m}\times J{A_{m}}^{*}\right)}\left(J^{2}-{A_{m}}^{2}v^{2}+2jA_{m}v\Omega -\Omega^{2}\right)$$ (9)

The generalization coefficients are obtained from the initial conditions Equation (2) and Equation (9):


$$\{\;\;\;\begin{array}{c} {}*20cC_{1mn}=\frac{D_{1mn}r_{1}-D_{2mn}A_{m}v}{r_{1}-r_{2}}\\ C_{2mn}=\frac{-D_{1mn}r_{2}+D_{2mn}A_{m}v}{r_{1}-r_{2}} \end{array}$$ (10)

The dynamic deflection function of the model is obtained by bringing Equation (6) to Equation (10) into Equation (3):


$$w(x,y,t)=\sum_{m=1}^{\infty }\sum_{n=1}^{\infty }[\;(D_{1mn}R_{1}\;+\;D_{2mn}\;A_{m}\;v\;R_{2})+D_{1mn}\;\cos(A_{m}v)\;t+\;D_{2mn}\;\sin(A_{m}v)\;t]\times \sin(A_{m}\;x)\;\sin(B_{n}\;y)$$ (11)

where $R_{1}=\frac{r_{1}e^{r_{2}t}-r_{2}e^{r_{1}t}}{r_{2}-r_{1}}$, $R_{2}=\frac{e^{r_{1}t}-e^{r_{2}t}}{r_{2}-r_{1}}$.

Specifically, when c = 0 and k = 0, the solution for the undamped vibration of the model can be obtained, i.e.,


$$w(x,y,t)=\frac{4P}{ab\rho h}\sum_{m=1}^{\infty }\sum_{n=1}^{\infty }[\frac{\text{1}}{J^{2}-{A_{m}}^{2}v^{2}}(\sin\left(A_{m}v\right)t-\frac{A_{m}v}{J})\times \sin\left(A_{m}x\right)\sin\left(B_{n}y\right)]$$ (12)

**Fig 3:**
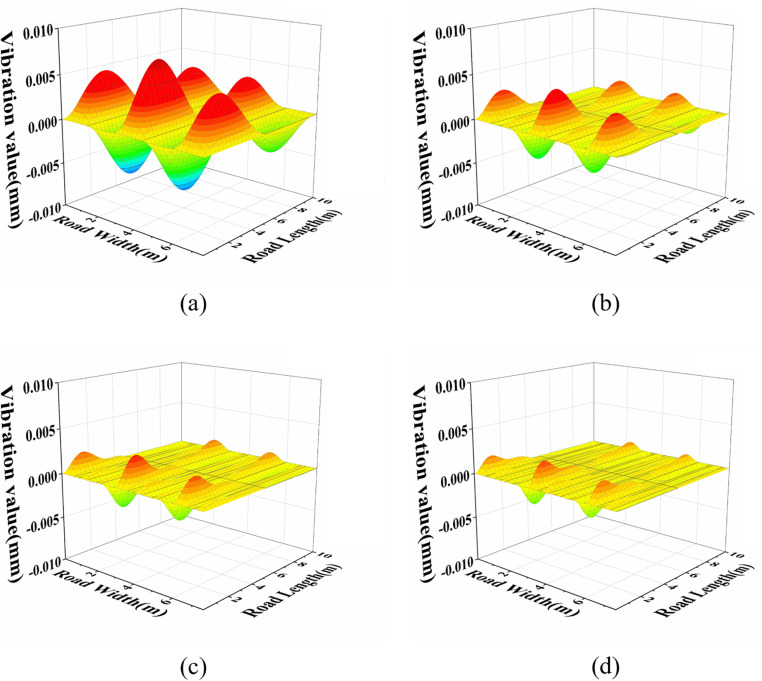
Semi-rigid base asphalt pavement vibration characteristics for different vibration patterns, (a) vibration pattern selected m = 1, n = 1, (b) vibration pattern selected m = 2, n = 2, (c) vibration pattern selected m = 3, n = 3, (d) vibration pattern selected m = 4, n = 4.

The form of the solution derived above aligns with those presented by Lin [41] and Yan [42], confirming the accuracy of the model and the solution for the semi-rigid base asphalt pavement four-sided cylinder-supported plate structure. Therefore, the vibration behaviour of the semi-rigid base asphalt pavement under different excitation conditions and vibration modes can be determined, as illustrated in Fig 3(a–d). During the excitation process, the vibration frequency is predominantly concentrated in the low-frequency range, with the external excitation frequency closely matching the intrinsic frequency of the road. To facilitate the analysis of road performance and structural optimization—particularly for improving subsequent simulation calculations [43,44]—the vibration mode selected for the study is *m* = 2, *n* = 2.

**Fig 4:**
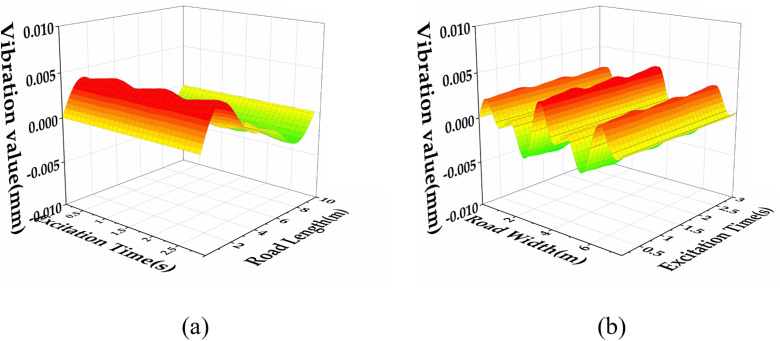
Semi-rigid base asphalt pavement data with different vibration times for the same vibration mode, (a) vibration data at different times in the width direction, (b) vibration data at different times in the length direction.

Based on the solution forms of Equations (11) and (12), the semi-rigid base asphalt pavement vibration state under a specific moving speed *v* exhibits periodic behaviours. In this study, the semi-rigid base asphalt pavement vibration model is analysed along both the width and length directions by selecting representative profiles. The vibration states corresponding to varying vibration times *t* are calculated, and the results are presented in Fig 4(a,b). These calculations demonstrate that both the width and length directions exhibit periodic motion over time under continuous excitation. This finding provides a solid theoretical foundation for further research.

#### DEVD-maxwell model.

Based on the Technical Specifications for Asphalt Pavement Design of Highways (JTGD50–2017) issued in China, the modeling parameters proposed by Pérez-Acebo [36] and Zu [37], and measured data from several highways and provincial semi-rigid base asphalt pavements in Henan Province, China, the primary simulation parameters for the road model were determined, as shown in Table 1.

**Table 1:** Selected road geometry and physical parameters.

Structural layer	Thickness of each layer (cm)	Width (m)	Modulus (MPa)	Poisson’s ratio	Density (kg/m^3^)
Asphalt concrete surface layer	18	8	11500*	0.25	2400
Semi-rigid base	40	8	18000**	0.25	2300
Lime stabilized soil base	20	8	6000**	0.25	1900
Soil base	150	8	90***	0.40	1900

*Dynamic modulus, test at 20 °C, with frequency of 10 Hz; **Resilience modulus; *** Resilience modulus of the top surface of the subgrade.

The reason for choosing the DEVD-Maxwell model is for balance computational efficiency, detection sensitivity, and potential applicability in analyzing semi-rigid base asphalt pavement debonding. In this study, a finite element model was constructed based on the approach proposed by Yan [29], using the physical parameters of the pavement structure (Table 1) and assuming a road length of 8 m in the direction of vehicle operation. However, the resulting model, composed of 73,034 cells and 78,732 nodes, introduced significant challenges in terms of computational storage and processing resources. Additionally, the low stiffness and large size of the soil base led to numerous unwanted local modes, which could interfere with the accuracy of the analysis.

Within classical plate-on-foundation formulations, the DEVD–Maxwell foundation is adopted here for its numerical efficiency and fidelity in road-structure simulation. Modeling the base as a spatially distributed spring–dashpot field with variable damping reduces the assembled degrees of freedom and memory while preserving the low-order bending modes of the pavement–base system. As a result, the assembled matrices are smaller and often better conditioned, which facilitates transient and steady-state solvers and enables parametric studies of debonding size, location, and moving-load speed at practical cost. Debonding is represented as a spatially localized reduction in interlayer stiffness (or added interface compliance), which the foundation can accommodate without remeshing the base domain. Modal comparisons (Table 2) show close agreement for the first modes relative to the reference model while using a smaller system, supporting the model’s accuracy-per-cost balance for the numerical analyses reported here.

**Table 2:** Comparison of different modal frequencies between the original model and DEVD- Maxwell.

Model	First-order mode frequency (Hz)	Second-order mode frequency (Hz)	Third-order mode frequency (Hz)	DOF (n)	Total runtime (s)	Peak memory (GB)
Original model	4.4787	5.6283	5.6283	518,403	222,103	30.03
DEVD- Maxwell	4.4778	5.2639	5.2639	317,884	161,440	22.41
Accuracy	0.020%	6.474%	6.474%	–	–	–

The specific equivalent treatment is as follows: since the first order mode is the overall subsidence of the road, the first three layers of the road have a large stiffness compared to the soil base, which can be regarded as a rigid body, and its mass is *M*. Assuming that there are n springs and m dampers, the stiffness of each spring is denoted as *ks*_*i*_ (*i* = 1, 2,..., n), and the equivalent stiffness of each damper at a particular displacement is denoted as *kd*_*j*_ (*j* = 1, 2,..., m) whose equivalent stiffness is:


$$K=\sum_{i=0}^{n}ks_{i}+\sum_{j=0}^{m}kd_{j}$$ (13)

**Fig 5:**
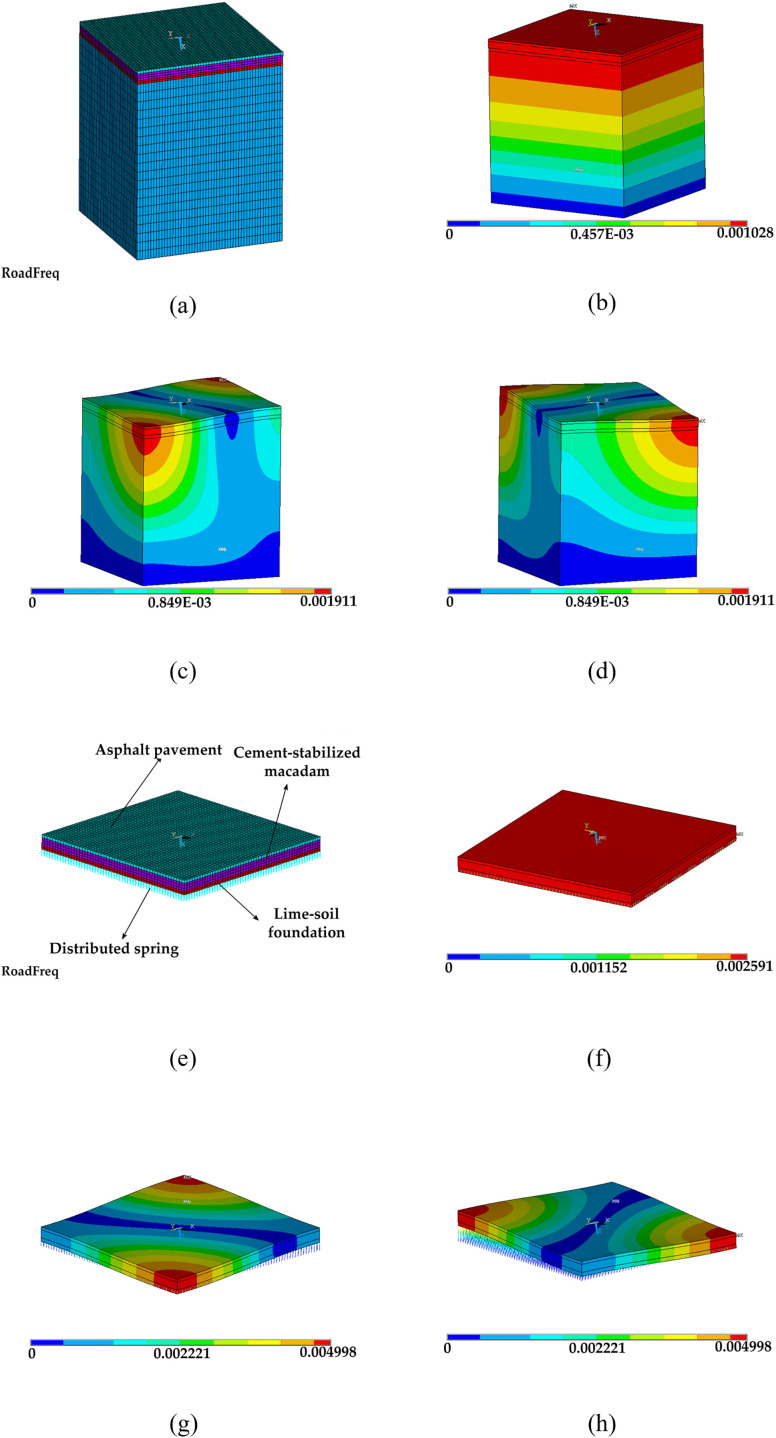
Comparison of original model and DEVD-Maxwell model road modes, (a) Original model, (b) First-order mode, (c) Second-order mode, (d) Third-order mode, (e) DEVD- Maxwell, (f) First-order mode of the DEVD- Maxwell, (g) Second-order mode of the DEVD- Maxwell, (h) Third-order mode of the DEVD- Maxwell.

This results in a first order frequency $f=\sqrt{K/M}$. The improved computational model is shown in Fig 5.

The spring stiffness *k* = 41426 N/m, connects each node and constrains all degrees of freedom at one end of the spring, while the first three degrees of freedom of the asphalt layer remain unchanged. Under the same excitation configuration, the modes are computed and the resulting frequencies—reported in **Table 2**—agree well with the reference solution. In particular, the DEVD–Maxwell formulation reproduces the first-order frequency within 0.020% of the original model while using fewer equations (degrees of freedom, DOF) and lower runtime and memory; see the added DOF (number of equations), Total runtime (s), and Peak memory (GB) columns in **Table 2**.

Relative to the original model, the DEVD–Maxwell formulation reduces the assembled number of equations (DOF) from 518,403–317,884 (−38.68%), shortens wall-clock runtime from 222,103 s to 161,440 s (−27.31%), and lowers peak memory from 30.03 GB to 22.41 GB (−25.37%), while keeping the first-mode frequency within 0.020% of the reference (second/third modes: 6.474%). Accuracy in Table 2 is computed asⅠ*f*_DEVD_ - *f*_Original_Ⅰ/ *f*_Original_ × 100%. These results indicate that the optimized foundation model maintains the required structural stiffness representation and low-order modal fidelity, with materially reduced computational cost, making it suitable for subsequent vibration and acoustic analyses under moving excitation.

**Fig 6:**
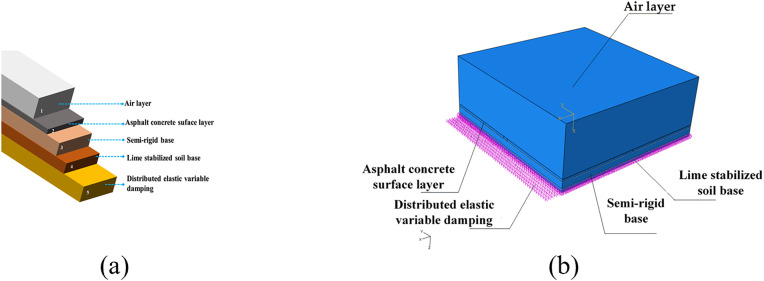
DEVD-Maxwell based acoustic-solid coupling modelling, (a) Pavement structure, (b) DEVD-Maxwell model structure.

In this study, road evaluation is primarily conducted by analyzing sound to assess the vibration characteristics of the pavement. The acoustic-solid coupling algorithm in ABAQUS is well-suited for addressing the coupling between vibration and sound. Consequently, ABAQUS is utilized as the analytical tool for the following calculations. The acoustic-solid coupling road analysis model, based on the DEVD-Maxwell approach, is illustrated in Fig 6. The model includes, from bottom to top, a distributed elastic variable damping, a lime stabilized soil base, a semi-rigid base, an asphalt concrete surface layer, and an air layer.

### Experimental setup

To verify the accuracy of the model and explore the correlation between sound pressure values and semi-rigid base asphalt pavement debonding, laboratory experiments were conducted using full-scale experimental specimen specifically designed and laid with distinct structural debonding.

The thickness of each layer in the full-scale experimental specimen was selected according to the structure shown in Table 1. From top to bottom, the layers consist of a 5 cm AC-16 asphalt concrete layer, a 10 cm AC-25 asphalt concrete layer, 40 cm of semi-rigid base paved in two layers, and a 30 cm lime soil base and road base. The specimen measures 2.3 m in length and 1.1 m in width, with the debonding position set at the center of the specimen and more than 0.35 m from the edges. The ratio of the debonding area to the non-debonding area is 7:3.

Moving point excitation was applied from one end of the specimen to the other under the same conditions as the simulation, using a 490 N excitation load. During the excitation process, nine detection points were set up. Detection points 1 and detection point 9 were positioned 35 cm from the left and right edges of the specimen, respectively, with an interval of 20 cm between adjacent detection points.

**Fig 7:**
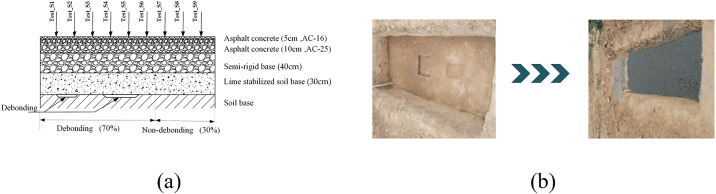
Full-scale specimen structure and field test, (a) Full-scale specimen structure drawing, (b) Full-scale specimen emptying location and field test.

The specimen contained two debonding regions, which were depressed basins. One had a height of 2 cm and a size of 20 cm × 40 cm, while the other had a depth of 2 cm and a size of 40 cm × 40 cm, separated by 50 cm. These details are illustrated in **Fig 7**.

During the data acquisition process, two observation points, P1 and P2, were established to collect sound data using pickups with a sampling frequency of 22.05 kHz. Observation point P1 was positioned 40 cm above the excitation source, while P2 was located 80 cm above the excitation source. The pickups moved in tandem with the moving excitation source throughout the experiment.

### Excitation scheme

**Fig 8:**
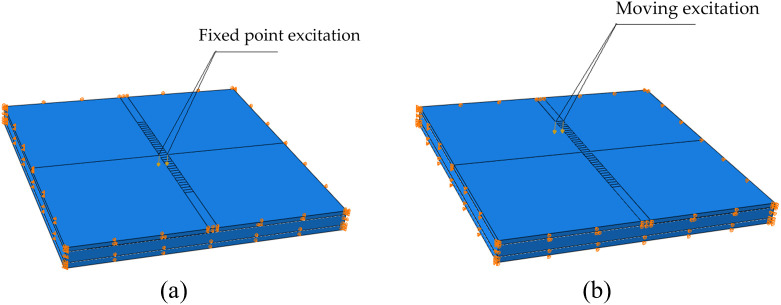
Incentive models and approaches, (a) fixed point excitation model, (b) moving excitation model.

Fixed-point excitation is more effective for inducing significant sound pressure changes in semi-rigid base asphalt pavement, making it suitable for obtaining maximum data, while continuous point excitation better aligns with the requirements for rapid semi-rigid base asphalt pavement detection. Accordingly, both excitation modes are configured for analysis. The fixed-point excitation is set with a duration of 1.0027 s and an excitation load of 490 N. Mobile excitation is simulated by applying the same load at different times and positions over a moving distance of 5.12 m and a duration of 1.707 s. The load model is illustrated in **Fig 8**.

### Evaluation indices and data processing

The volume of sound is characterized by the amplitude, frequency, and phase of sound pressure, which are parameters dependent on both space and time. To quantify sound volume more accurately, the Sound Pressure Level (SPL) is commonly used to represent acoustic values [45]. However, because the human auditory system is frequency-selective, SPL alone does not fully capture how humans perceive attenuated sounds. To address this, the A-weighted sound pressure level is widely employed. This measurement accounts for the frequency response characteristics of the human ear and auditory system, providing a more accurate representation of perceived sound [46].

Calculate the A-weighted sound pressure level *Ap*_*i*_ under the frequency condition at point *i* according to the sound pressure spectrogram, and the calculation formula is shown in Equation 14:


$$Ap_{i}=20\times \mathrm{lg}\left(\frac{p_{i}(e)}{p(ref)}\right)+p_{i}(amend)$$ (14)

where: *Ap*_*i*_ is the A-weighted sound pressure level at the *i*th point; *i* is the frequency at the *i*th point; p_*i*_(e) is the sound pressure value at the *i*th frequency; p(ref) is the reference sound pressure, which is taken as 2 × 10^−5^ Pa; and p_*i*_(amend) is the A-weighted correction value as shown in Equation 15:


$$R_{a}(f)=\frac{{f_{d}}^{2}f^{4}}{\left(f^{2}+{f_{a}}^{2}\right)\left(f^{2}+{f_{d}}^{2}\right){\left(f^{2}+{f_{b}}^{2}\right)}^{0.5}{\left(f^{2}+{f_{c}}^{2}\right)}^{0.5}}$$ (15)

$$p_{i}(amend)=2.0+20\times \mathrm{lg}(R_{a}(f))$$ (16)

where *f* is the calculated frequency, *f*_*a*_ = 20.6 Hz, *f*_*b*_ = 107.7 Hz, *f*_*c*_ = 737.9 Hz, *f*_*d*_ = 12200 Hz.

Using the results of Equation 14, the total sound pressure level $\overset{―}{Ap_{i}}$ and total sound pressure level *Ap* at point *i* of the sound pressure are calculated as shown in Equations 17 and 18, respectively.


$$\overset{―}{Ap_{i}}=10\times lg(\sum_{i=1}^{i}10^{\frac{Ap_{i}}{10}})$$ (17)

$$Ap=10\times lg(\sum_{i=1}^{N}10^{\frac{Ap_{i}}{10}})$$ (18)

## Results

We first report numerical results, then experimental results, followed by a direct simulation–experiment alignment and the acoustic–GPR performance analysis.

### Model simulation result

This study focuses on semi-rigid base asphalt pavement using the DEVD-Maxwell model to evaluate the acoustic characteristics of pavements through acoustic-solid coupling and A-weighted sound pressure level methods, with the goal of inferring pavement debonding conditions. The research is divided into two core components: first, model simulations are used to analyse the performance of non-debonding pavements under fixed-point and mobile excitation, providing a detailed comparison of these excitation modes. Secondly, building on these findings, mobile excitation—better suited for field testing—is utilized to further explore the mathematical relationships between debonding areas and evaluation indices, as well as the interrelationship among evaluation indices.

#### Road non-debonding modelling calculations.

**Fig 9:**
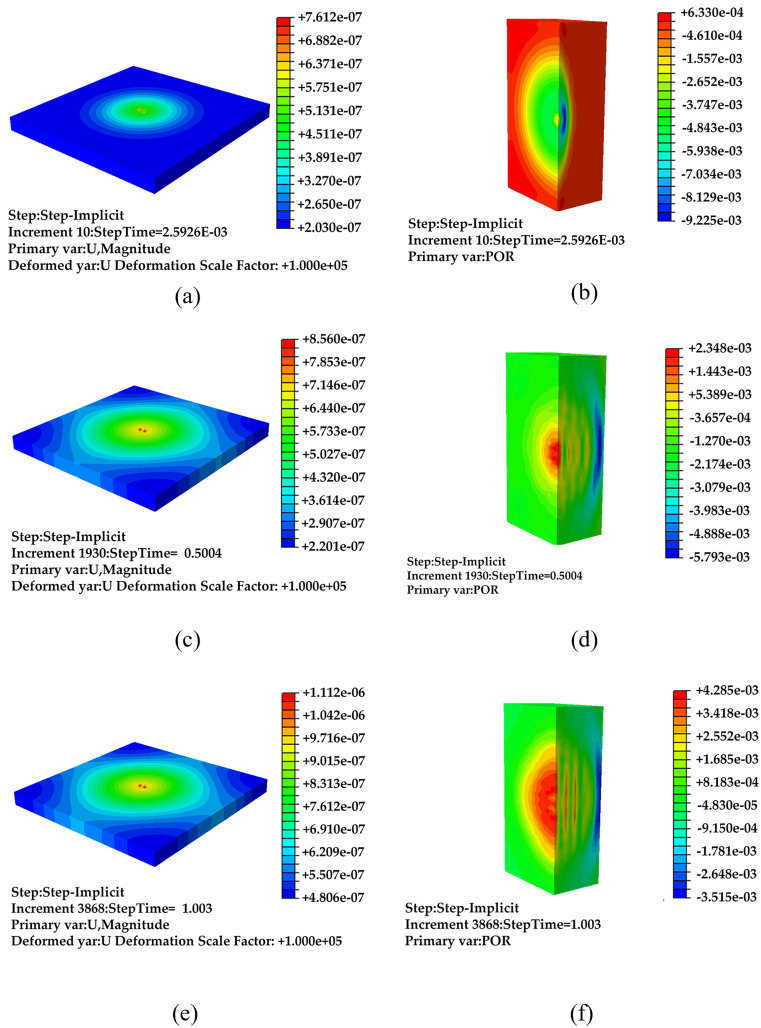
Simulation results of fixed-point excitation under non-debonding conditions, (a) t = 0.0026 s vibration data, (b) t = 0.0026 s sound data, (c) t = 0.55 s vibration data, (d) t = 0.55 s sound data, (e) t = 1.1 s vibration data, (f) t = 1.1 s sound data.

Fixed-point incentives: The fixed excitation point is located at the center of the model, as shown in Fig 8a. As the excitation vibration propagates through the pavement and transmits into the air via acoustic-solid coupling, the sound emitted by the pavement is captured. The variation in pavement vibration over time (*t*) is analysed in Fig 4a and Fig 4b. For this simulation, data at three specific time points are examined within the time domain: *t* = 0.0026 s (Fig 9a and 9b), *t* = 0.55 s (Fig 9c and Fig 9d), and *t* = 1.1 s (Fig 9e and Fig 9f). In Fig 9, panels a, c, and e illustrate the simulated vibration under fixed-point excitation on the pavement, while panels b, d, and f depict the corresponding sound pressure values within the air layer. For subsequent analyses, the 1/2 field model will continue to be used.

**Fig 10:**
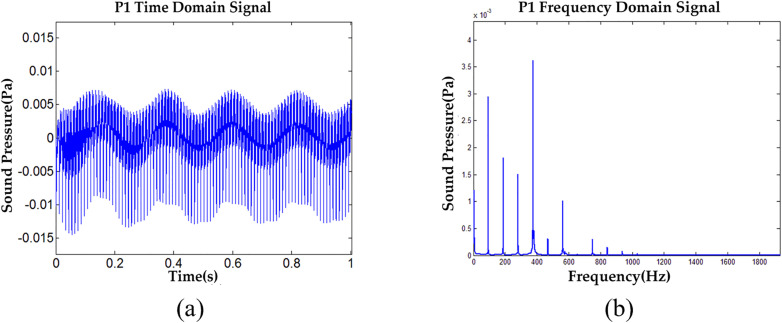
VTDD and VFDD data for Test Point 1 under non-debonding and fixed-point excitation conditions, (a) P1 VTDD, (b) P1 VFDD.

Five observation points in the sound field generated by the road excitation were selected to measure sound pressure. Their node numbers are 5122, 4238, 26703, 26807, and 26963, with corresponding coordinates of (0, 0, 0), (3.05, 0, 0), (0, 0, –0.689), (0, 0, –1.267), and (0, 0, –2.133). All observation points are located above the road, with Observation Point 1 serving as the excitation point (this applies in subsequent descriptions as well). Fig 10 presents the simulation results for Voice Time-Domain Data (VTDD). The corresponding Vibration Frequency-Domain Data (VFDD) are also analyzed for the same observation points, where Fig 10a through 10b depict the time-domain signals recorded at Observation Points 1 (points 2–5 are not shown here).

**Fig 11:**
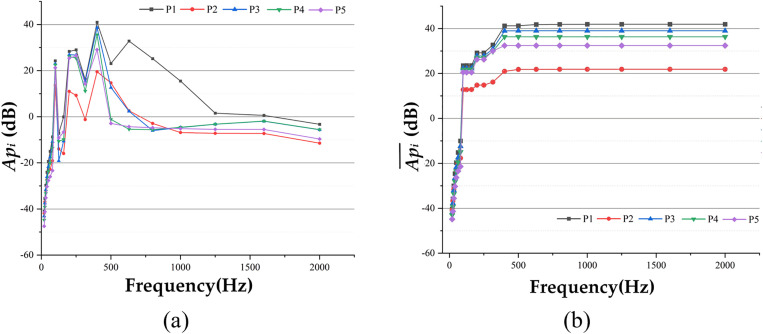
Comparison of data at each observation point under fixed excitation conditions, (a) Api Data Comparison, (b) $\overset{―}{Ap_{i}}$ Data Comparison.

The sound pressure time domain data from the five observation points were extracted and analyzed using spectral analysis. Equations (14) and (17) were applied to calculate the *Ap*_*i*_ and $\overset{―}{Ap_{i}}$ of each observation point and, with the results shown in Fig 11. Additionally, Equation (18) was used to calculate the overall *Ap* values for the observation points, which are 42.2498 dB, 22.2252 dB, 39.3342 dB, 36.6387 dB, and 32.4875 dB, respectively.

An in-depth analysis of the results presented in Fig 11 reveals that the system response can be interpreted as a superimposed effect of multiple harmonic excitations, owing to the periodic nature of the applied loads. Examination of the response in both the time and frequency domains clearly demonstrates a direct correspondence between the individual peak frequencies and the harmonic excitation frequencies of each order, confirming the accuracy and reliability of the calculations. Further analysis of the frequency spectrum shows that the primary energy contribution of *Ap*_*i*_ and $\overset{―}{Ap_{i}}$ lies within the frequency range below 475 Hz, with negligible contributions beyond this range. This indicates that the excitation energy from fixed-point excitation on the non-debonding pavement surface is concentrated in the low-frequency range below 475 Hz. This energy distribution characteristic explains the relatively low noise level of the road surface after A-weighting, providing robust data support for subsequent optimization efforts.

**Fig 12:**
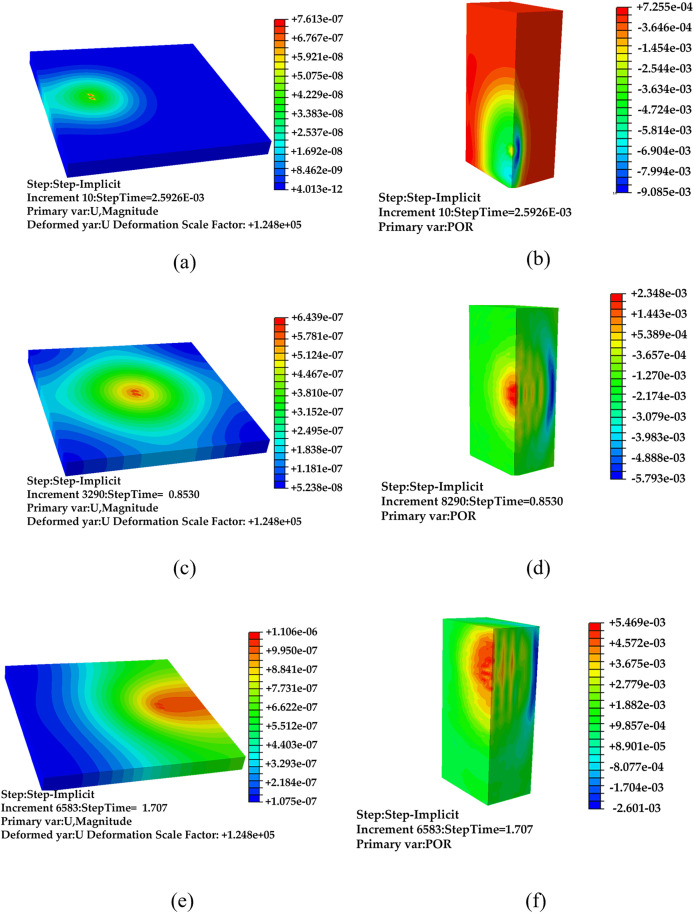
Simulation results of moving point excitation under non-debonding condition, (a) t = 0.0026 s vibration data, (b) t = 0.0026 s sound data, (c) t = 0.55 s vibration data, (d) t = 0.55 s sound data, (e) t = 1.1 s vibration data, (f) t = 1.1 s sound data.

Mobile point incentive: The moving point excitation mode, depicted in Fig 8b, is configured with an excitation speed of v = 4.5 m/s. The observation moments are selected as t = 0.0026 s, 0.55 s, and 1.1 s in the time domain. The simulation results for road vibration and sound (1/2 distribution) at these moments are presented in Fig 12a to Fig 12f.

**Fig 13:**
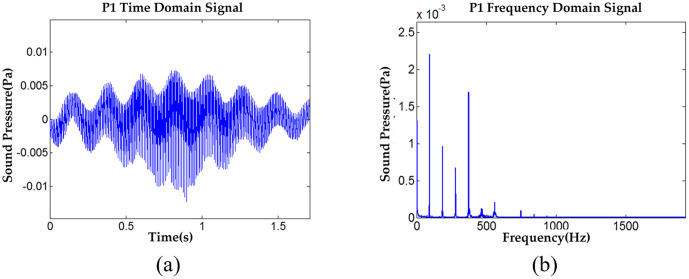
VTDD and VFDD data for Test Point 1 of non-debonding under moving point excitation conditions, (a) P1 VTDD, (b) P1 VFDD.

Under moving point excitation conditions, the VTDD and VFDD data for observation point 1 are sequentially presented in Fig 13a to Fig 13b (points 2–5 are not shown here).

**Fig 14:**
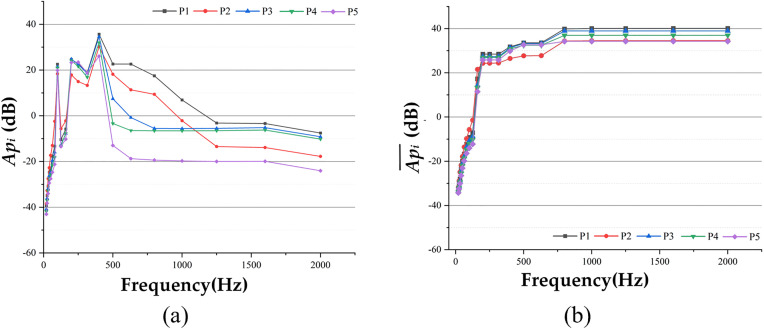
Comparison of data for each observation point under mobile excitation conditions, (a) Api Data Comparison, (b) $\overset{―}{Ap_{i}}$ Data Comparison.

Based on the sound pressure signal and spectral data at each observation point, its *Ap*_*i*_ and $\overset{―}{Ap_{i}}$, which are shown in Fig 14, are calculated according to Equation (14) and Equation (17).

The *Ap* values for the five observation points were calculated using Equation (18) and are as follows: 36.8544 dB, 31.2313 dB, 35.5421 dB, 33.2724 dB, and 30.0518 dB.

Based on the calculation results, it is concluded that the sound pressure under mobile loading is approximately 2 dB lower than that under fixed loading, with the output energy primarily concentrated below 750 Hz. While there are differences between the results of mobile and fixed loading, the mobile load demonstrates significant potential for future application and offers greater flexibility during the continuous detection process of semi-rigid base asphalt pavement. More importantly, it provides a robust foundation for the continuous detection of semi-rigid base asphalt pavement debonding. Therefore, this study primarily adopts the moving-point excitation method to conduct an in-depth analysis of the acoustic characteristics of semi-rigid base asphalt pavement, aiming to enhance the accuracy and rigor of the analysis.

#### Road presence debonding model calculations.

**Fig 15:**
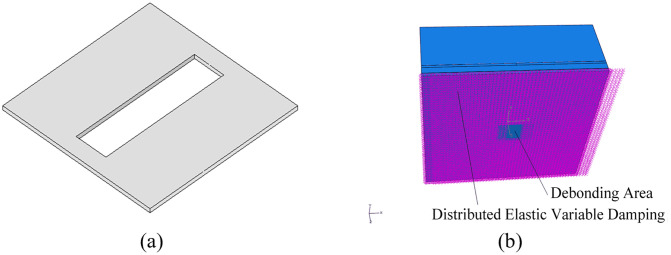
Debonding Model Simulation Settings, (a) debonding Model (1.5m × 4m), (b) DEVD-Maxwell debonding model.

A debonding model is established between the stabilized gravel layer and the roadbed within the semi-rigid base asphalt pavement structure. The model calculates the impact of varying debonding areas, which are set to five specifications: 1 m × 4 m, 1.5 m × 4 m, 2 m × 4 m, 3 m × 4 m and 4 m × 4 m. The model is illustrated in Fig 15a and Fig 15b. To improve efficiency, data are collected from two observation points with coordinates (0, 0, 0) and (0, 0, −0.4), respectively.

**Fig 16:**
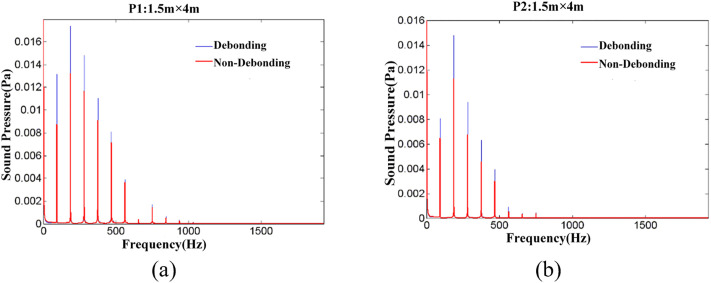
Comparison of Debonding Model and Non-debonding Model Simulation Data, (a) P1(1.5m × 4m), (b) P2(1.5m × 4m).

Based on the simulation results, the VFDD data for observation points P1 (1.5m × 4m) and P2 (1.5m × 4m) under debonding and non-debonding conditions are presented in Fig 16.

**Fig 17:**
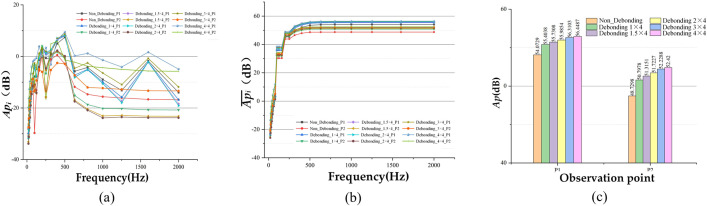
Comparison of the data of each observation point under different conditions of desiccation debonding, (a) Api Data Comparison, (b) $\overset{―}{Ap_{i}}$ Data Comparison, (c) Ap Data Comparison.

Under the given simulation conditions, the sound pressure spectral data for each observation point were calculated for various debonding areas. The resulting *Ap*_*i*_, $\overset{―}{Ap_{i}}$ and *Ap* data pairs for different observation points are presented in Fig 17.

From the calculations in Fig 17, it can be seen that:

(1) As the debonding area increases, the overall trend of the Api data curves remains consistent across observation points, particularly for the same observation point. Overall, the curve for observation point P1 is higher than that for observation point P2, and the curves shift upward with increasing debonding area.
(2) Under the fixed excitation conditions, for both non-debonding and varying debonding area scenarios, *Ap* reaches more than 95% of the $\overset{―}{Ap_{i}}$ value at *i* = 750 Hz. The excitation sound pressure values are primarily concentrated within the 750 Hz frequency range.
(3) By comparing the total sound pressure value reveals that *Ap* increases with the debonding area, exhibiting a nonlinear relationship. On average, the *Ap* value at observation point P1 is 4.28 dB higher than at observation point P2.

### Experimental validation

**Fig 18:**
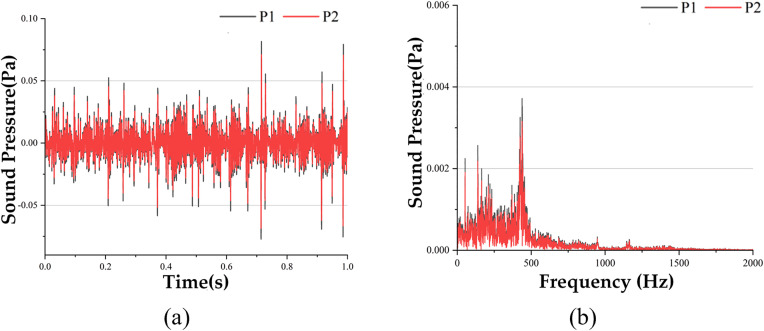
Comparison of VTDD and VFDD at different checkpoints P1 and P2 of the full-scale specimen, (a) Test_s1 VTDD, (b) test_s1 VFDD.

Using the above method, this study collected VTDD and VFDD at test points 1 through 9 for observation points P1 and P2 under moving excitation conditions, as shown in Fig 7. The results are presented in Fig 18 (test points 2–9 are not shown here).

**Fig 19:**
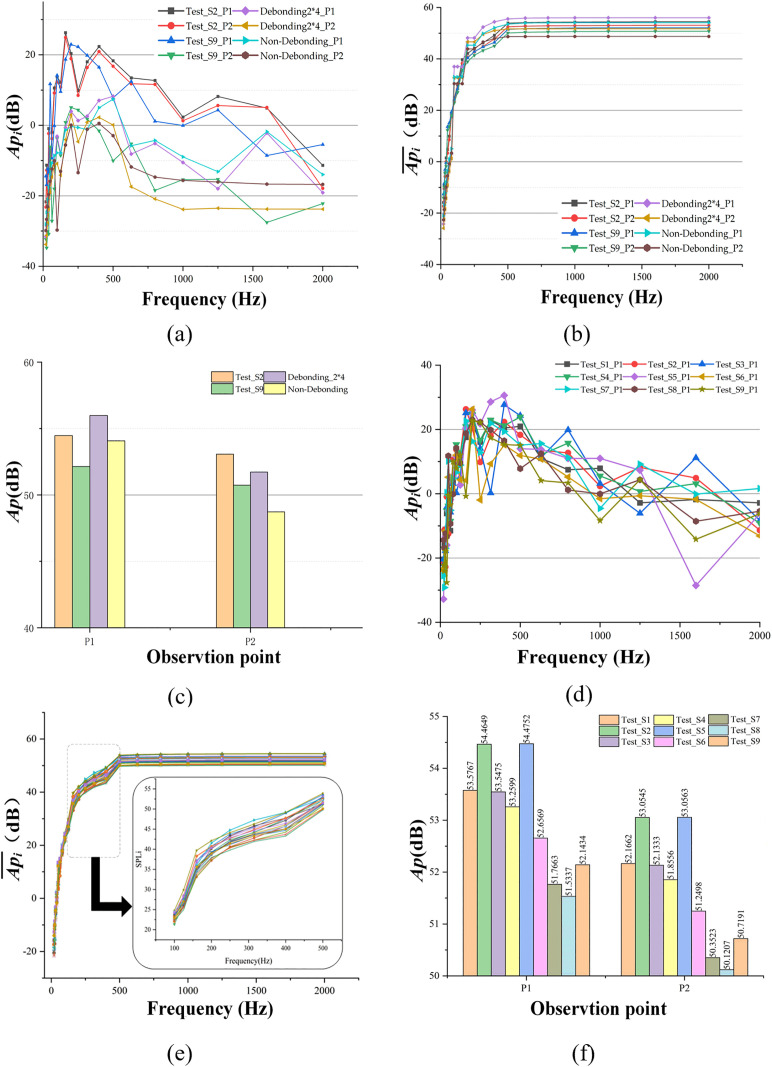
Test data and comparative analysis of full-scale specimen, (a) Model and Test Api data, (b) Model and Test $\overset{―}{Ap_{i}}$ data, (c) Model and Test Ap data, (d) Test Api data, (e) Test $\overset{―}{Ap_{i}}$ data, (f) Test Ap data.

Based on the data in Fig 18, the data of *Ap*_*i*_, $\overset{―}{Ap_{i}}$ and *Ap* at each detection point are obtained and compared with the simulation data as shown in Fig 19a to Fig 19f.

#### Comparative analysis of single point test results and models.

In this experiment, the sound data from two locations, detection point 2 (debonding location) and detection point 9 (non-debonding location). These data were compared with simulation results for a debonding area of 2 m × 4 m and non-debonding conditions in the computational model. The *Ap*_*i*_, $\overset{―}{Ap_{i}}$ and *Ap* data patterns were analyzed, and the results are shown in Fig 19a to 19c.

(4) From the frequency domain data reveals that the energy of both the simulation and experimental data is primarily concentrated within 500 Hz. In the simulation model, energy is predominantly distributed at 97 Hz, 189 Hz, 283 Hz, 377 Hz, and 471 Hz, with minimal contributions from *Ap*_*i*_ to the overall *Ap* at other frequencies. In contrast, the experimental data exhibit a peak energy value at 470 Hz, but the frequency band is very narrow. Additionally, the energy variation within 500 Hz remains within one order of magnitude across other frequencies, resulting in a relatively balanced contribution of *Api* to the overall Ap within this range.
(5) Comparison of simulation and experimental data indicates that at the non-debonding position, the simulation model produces an average sound pressure value 1.9 dB higher than that of the full-scale experimental specimen. At the debonding position, the simulation model’s average value is 1.3 dB higher. This discrepancy is attributed to differences in the moduli between the simulation model and the full-scale experimental specimen.
(6) In a further assessment of the simulation and experimental data, the VTDD of inspection points 2 and 9, along with their corresponding VFDD, exhibit consistent frequency distributions between the debonding and non-debonding simulation data. Furthermore, the frequency distributions of the simulation data align with those of the full-scale experimental specimen. Additionally, when comparing the trends and performance of *Ap*_*i*_, $\overset{―}{Ap_{i}}$ and *Ap*, the responses of the simulation model and the full-scale experimental specimen are fundamentally consistent, thereby validating the accuracy of the simulation model.

#### Analysis of excitation sound data from full-scale experimental specimen.

In this experiment the *Ap*_*i*_, $\overset{―}{Ap_{i}}$ and *Ap* of P1 and P9 of the 9 test points were analysed as shown in Fig 19d to Fig 19f, which were analysed and learnt:

(7) Analysis of *Ap*_*i*_ and $\overset{―}{Ap_{i}}$ trends shows that the curves for all nine detection points follow the same pattern across frequencies. The *Ap* values are primarily concentrated within the 100 Hz to 500 Hz range, consistent with the simulation model. This finding confirms the energy distribution of the sound pressure detected in the semi-rigid base asphalt pavement structure and aligns with the results from the full-scale experimental specimen.
(8) Analysis of *Ap* differences (Fig 19d) reveals that detection point 8, located at the center of the non-debonding test area, exhibits the smallest *Ap* value. Using detection point 8 as a reference, the differences △*Ap*_P*i*|P8, for other detection points are calculated as follows: △*Ap*_P1|P8 = 2.043 dB; △*Ap*_P2|P8 = 2.931 dB; △*Ap*_P3|P8 = 2.013 dB; ∆*Ap*_P4|P8 = 1.762 dB; ∆*Ap*_P5|P8 = 3.241 dB; ∆*Ap*_P6|P8 = 1.123 dB; ∆*Ap*_P7|P8 = 0.232 dB; ∆*Ap*_P9|P8 = 0.610 dB.
(9) Comparing the debonding dimensions of the full-scale experimental specimen and the locations of the detection points, it is concluded that the *Ap* value is highest for the 40 cm × 40 cm debonding area, followed by the 20 cm × 40 cm debonding area, with a difference of 0.30 dB. The *Ap* values near the debonding locations are consistently higher than those at non-debonding locations, exhibiting distinct *Ap* characteristics. This observation supports the use of *Ap* as a reliable basis for distinguishing between semi-rigid base asphalt pavement debonding and non-debonding areas.

These results confirm that the mobile detection method effectively measures semi-rigid base asphalt pavement sound pressure and calculates *Ap*, making it a viable technique for detecting debonding in semi-rigid base asphalt pavements.

## Discussion

### Acoustic vs. GPR performance

To further evaluate the reliability of the proposed method and its advantages over existing technologies, this study conducted a comparative experiment using GPR, a widely employed non-destructive testing technique for pavement distress detection [7,10].

**Fig 20:**
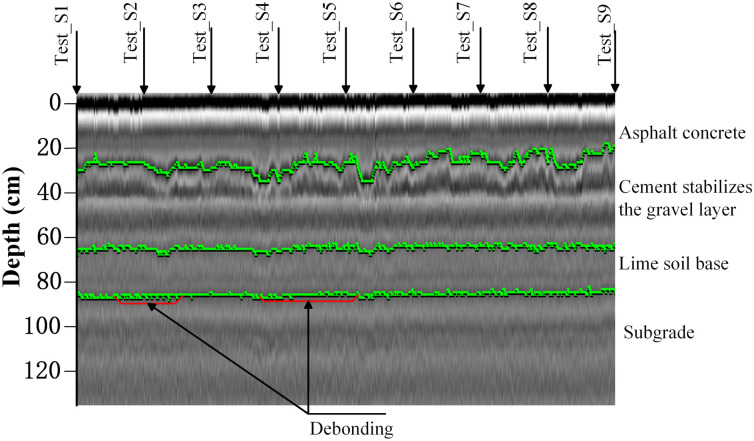
GPR in Full-scale specimen detection data.

In the experiment, a 2 GHz GPR antenna was utilized to scan the full-scale specimen. As shown in Fig 20, the GPR method successfully identified the interface boundaries between the asphalt concrete, semi-rigid base, lime-stabilized soil base, and soil base. However, it failed to detect the two pre-set debonding areas within the specimen. These results indicate that despite using a 2 GHz high-frequency antenna with advanced processing, failed to detect the pre-set debonding areas in the full-scale specimen, highlighting its limitation in identifying thin-layer interlayer debonding in semi-rigid base asphalt pavements. This limitation is primarily due to three key factors: minimal dielectric contrast, resolution constraints, and signal attenuation. First of all, the dielectric properties of the semi-rigid base asphalt pavement and its underlying layers exhibit only slight variations, resulting in weak electromagnetic reflections that make debonding areas indistinguishable. Secondly, while high-frequency GPR enhances lateral resolution, its ability to resolve fine-scale delamination’s remains limited, particularly in complex multi-layered pavement structures where reflections from adjacent interfaces may obscure debonding signals. Finally, signal attenuation and noise interference, exacerbated by the heterogeneity of asphalt mixtures and potential moisture presence, further reduce detection accuracy.

In principle, increasing the center frequency of GPR shortens the electromagnetic wavelength in asphalt (e.g., at 2 GHz the wavelength is on the order of several centimeters for εr ≈ 4–6), which can improve vertical resolution for thin interfaces. However, pushing to 3–4 GHz or beyond brings significant attenuation, shallower penetration depth, stronger sensitivity to moisture/heterogeneity, and a reduced signal-to-noise margin. For millimeter-scale debonding, even very-high-frequency antennas may still face resolution and contrast limits in practice, particularly on in-service pavements with variable conditions. Consequently, while higher-frequency GPR can offer theoretical resolution gains, its field utility for very thin interlayers remains conditional on site-specific constraints.

Why acoustics is more sensitive to sub-wavelength thin-layer debonding. In semi-rigid base asphalt pavements, a thin interlayer debonding—even on the order of 1 mm, far below the electromagnetic wavelength—changes the effective boundary condition and interlayer stiffness of the plate-on-foundation system. Under stationary impact or moving excitation, this local compliance perturbation alters the low-order bending response, producing small downshifts in modal frequency and increases in local vibration amplitude/curvature. The radiated sound therefore redistributes toward the low–mid frequency band most relevant for roadway acoustics. Because the A-weighted SPL indices aggregate energy in this perceptually weighted band, they exhibit consistent, measurable changes near debonded zones, as confirmed by our simulation and full-scale results. By contrast, GPR relies on dielectric contrast and wavelength-limited vertical resolution; when the separation is millimeter-scale, the electromagnetic return may remain below resolvable contrast even with advanced processing, explaining the observed miss on preset thin layers.

These findings confirm that even with high-frequency excitation and advanced processing techniques, GPR is not well-suited for detecting small-scale interlayer debonding in semi-rigid base asphalt pavements, underscoring the need for more effective alternative methods.

### Research gap and future directions

While the acoustic-based detection method has proven more effective than GPR in detecting thin-layer interlayer debonding in semi-rigid base asphalt pavements, certain challenges remain. Future research should explore integrating acoustic and GPR method**s** to develop a multi-modal detection framework, leveraging GPR’s strength in identifying large-scale subsurface anomalie**s** and the acoustic method’s higher sensitivity to thin-layer defects. Additionally, optimizing excitation mechanisms and incorporating machine learning-based signal analysis could further enhance detection accuracy and automation.

Although validated on a full-scale specimen, further real-world testing is required to evaluate this method under varying traffic and environmental conditions. In real-world deployment, two issues primarily constrain scalability: ambient noise/SNR and excitation variability. First, the acoustic response is embedded in a complex noise field (tire–road interaction, powertrain/aerodynamic noise, cross-traffic, wind), which depresses and fluctuates the signal-to-noise ratio (SNR) across speed, surface texture, moisture, and temperature; this directly impacts the detectability of thin interlayers and the stability of the A-weighted indices. Second, under moving or continuous excitation, the excitation spectrum and energy depend on speed, axle load, tire tread, and contact dynamics; these source variations reshape measured amplitudes and spectra and can confound thin-layer indicators when not separated from structural response. Consequently, transferability from laboratory to roadway hinges on characterizing both the ambient-noise floor (and its variability) and excitation variability; otherwise, thresholds and comparisons across sites or campaigns may be biased. Detailed acquisition or processing protocols are beyond the scope of this study and will be addressed in future work.

This study does not include in-service traffic or environmental field data; we will collect and analyze such data in forthcoming work to establish operational performance and thresholds. Future efforts should focus on adapting it for automated pavement monitoring systems, enabling intelligent infrastructure maintenance with improved efficiency and reliability. Addressing these gaps will strengthen the method’s practicality for large-scale deployment in pavement health assessments.

## Conclusions and future work

In this study, we investigated the sound characteristics of semi-rigid base asphalt pavement debonding using an efficient DEVD-Maxwell model coupled with acoustic–solid interaction. On this basis, we constructed a simplified DEVD-Maxwell framework suitable for acoustic evaluation and implemented it in ABAQUS, providing the modeling foundation for subsequent analysis.

We introduced A-weighted sound-pressure-level indices (*Ap*_*i*_, $\overset{―}{Ap_{i}}$, *Ap*) as interpretable acoustic metrics. Considering different detection modes and speeds, the acoustic energy consistently concentrates in the low–mid frequency band, and the indices distinguish intact from debonded conditions from both local (point-wise) and overall perspectives, highlighting their utility for thin-layer debonding evaluation.

This study does not propose a new vibration theory. The novelty lies in an application-oriented integration: a computationally tractable DEVD–Maxwell foundation coupled with acoustic–solid interaction and A-weighted SPL indices, validated at full scale. Full-scale mobile experiments showed that *Ap* is elevated near debonding locations, and these signatures can serve as reliable indicators of interlayer debonding. Furthermore, a head-to-head comparison with GPR demonstrated the superior sensitivity of the proposed acoustic method to thin-layer debonding under identical conditions—which GPR failed to identify, indicating promise as a robust tool for pavement health assessment and road maintenance.

A comparative analysis of fixed-point and moving-point detection methods demonstrated that moving-point detection appears more practical for future real-road deployment; however, in-service validation is still required. Future research will focus on expanding the detection range, optimizing the model, and enhancing detection efficiency to further improve the application value of semi-rigid base asphalt pavement debonding detection.

## Supporting information

S1 File (RAR)
